# Investigation of the Effect of Oxide Additives on the Band Gap and Photocatalytic Efficiency of TiO_2_ as a Fixed Film

**DOI:** 10.3390/ma17184671

**Published:** 2024-09-23

**Authors:** Mabrouka Ghiloufi, Tobias Schnabel, Simon Mehling, Salah Kouass

**Affiliations:** 1 Laboratory of Materials: Treatment and Analysis, National Institute of Research and Physico-Chemical Analysis, Faculty of Sciences of Bizerte, Carthage University, Ariana 2020, Tunisia; mabrouka.ghiloufi@fsb.ucar.tn (M.G.); koissa2000@yahoo.fr (S.K.); 2 Research Group “Photonics and Water”, Institute for Sustainable Water Systems, Hof University of Applied Sciences, 95028 Hof, Germany; simon.mehling@hof-university.de

**Keywords:** photocatalysis, micropollutants, titanium dioxide

## Abstract

The effects of various additives (Y_2_O_3_, Ga_2_O_3_, and WO_3_) on photocatalytic degradation efficiency under UV light-emitting diodes (LEDs) and the optical properties of TiO_2_ Degussa P25 were investigated using ketoprofen and diclofenac, two non-steroidal anti-inflammatory drugs commonly detected in German rivers. Experimental results demonstrated that thin films containing these additives exhibited similar photocatalytic degradation efficiencies as pure TiO_2_, achieving a 30% degradation of ketoprofen over 150 min. In contrast, the Y_2_O_3_/TiO_2_ thin film showed significantly improved performance, achieving a 46% degradation of ketoprofen in 180 min. Notably, the Y_2_O_3_/TiO_2_ system was three times more effective in degrading diclofenac compared to pure TiO_2_. Additionally, the Y_2_O_3_/TiO_2_ photocatalyst retained its activity over three successive cycles with only a slight decrease in efficiency. The photocatalytic degradation of both organic pollutants followed first-order kinetics with all photocatalysts. The investigation included SEM imaging to assess the surface homogeneity of the thin films and UV-vis solid-state spectroscopy to evaluate the impact of the additives on the energy band gap of TiO_2_.

## 1. Introduction

Water pollution caused by the production and consumption of pharmaceutical molecules is a significant environmental threat [1]. Prescription and non-prescription drugs are often used excessively for minor ailments without emergency cases [2]. These drugs encompass a wide range, including anti-inflammatories, analgesics, antibiotics, and anti-stress medications. Non-steroidal anti-inflammatory drugs (NSAIDs) such as ketoprofen and diclofenac are analgesic compounds that have been widely detected with concentrations exceeding 1 µg/L in both influent and effluent of wastewater treatment plants (WWTPs) and reach 28.4 µg/L for diclofenac and 260 µg/L for ketoprofen in aquatic environment [3,4]. This indicates that conventional wastewater treatment methods are insufficient to eliminate NSAIDs from wastewater [5]. This widespread presence is attributed to excessive use, with annual consumption of active ingredients of drugs exceeding 300 mg per inhabitant in western Europe [6] with 100 tons per year for diclofenac in many countries [7]. Previous studies have demonstrated the significant toxicity of ketoprofen when exposed to sunlight, resulting in highly toxic photoproducts [8]. Similarly, the toxicity of diclofenac in WWTPs and surface waters has been shown to affect aquatic life adversely and indirectly harm human health [9,10,11].

Various processes have been employed in wastewater treatment, including photolysis [12], adsorption [13], ozonation, sonolysis [13], activated carbon [14], and photocatalysis [3,15]. Among these, photocatalysis is the most effective advanced oxidation process (AOP) for degrading organic pollutants, being eco-friendly, sustainable, and cost-effective [3,16,17]. The photocatalytic degradation of pharmaceutical pollutants is driven by a reaction between the organic contaminant molecules and reactive species such as the superoxide radical ($O_{2}^{.-}$) or hydroxyl radical (${OH}^{.}$), leading to their degradation into CO_2_ and H_2_O [18]:

$$Organic\;contaminant+O_{2}^{.-}/{OH}^{.}\to {CO}_{2}+H_{2}O$$

Titanium dioxide (TiO_2_) is a well-known semiconductor photocatalyst extensively studied for its photocatalytic properties under UV light, attributable to its energy gap of approximately 3.23 eV [19]. TiO_2_’s stability, its highly reactivity to generate radicals (O_2_·⁻ and OH·) under UV light, and its low cost makes it a preferred photocatalyst. Numerous studies have investigated its use in degrading dyes [20,21,22] and pharmaceutical pollutants [23,24,25]. Efforts to enhance TiO_2_’s efficiency under UV light or to extend its reactivity into the visible spectrum have involved doping and association processes, aiming to utilize sunlight as a sustainable energy source [3,26,27,28].

In this study, photocatalysis was selected as the AOP to degrade two prominent NSAIDs, ketoprofen (KETO) and diclofenac (DICLO), using TiO_2_. Subsequently, the effect of various additives (WO_3_, Y_2_O_3_, and Ga_2_O_3_) on the photocatalytic properties of TiO_2_ was examined. These properties include homogeneity of the photocatalysts as thin films, photocurrent, energy gap, and photocatalytic efficiency. The photocatalysts were immobilized on FTO glass, facilitating their reuse without material loss in treated water. This investigation employed a custom-designed small photoreactor with fixed parameters, developed by colleagues in the laboratory to study photocatalysis and photocurrent simultaneously. The design aims to be cost-effective and energy-efficient. UV light-emitting diodes (UV-LEDs) were chosen as the light source due to their advantages over mercury lamps, including the absence of mercury toxicity, low voltage operation, rapid startup, compact size, longer lifespan, and, most importantly, because LED can generate a specific wavelength that is near to the used photocatalyst’s wavelength adsorption [29,30,31,32].

This study addresses the gap in the literature by providing new insights into the enhanced photocatalytic degradation of NSAIDs using TiO_2_ with various additives. It also explores the practical application of immobilized photocatalysts and UV-LED technology in an eco-friendly, cost-effective system.

## 2. Methods

### 2.1. Chemicals and Materials

The photocatalysts are prepared using the ductile blade method from a titanium isopropoxide (TTIP, Sigma Aldrich, St. Louis, MO, USA) based sol-gel. The first step is to prepare a solution to form a TiO_2_ sol-gel as the base. This involves mixing of deionized water, TTIP, HNO_3_ (≥65°), acetyl acetate, and isopropanol (Carl Roth, Karlsruhe, Germany). All these precursors are combined in the same reactor equipped with a magnetic stirrer for 1 h. In the second step, the photocatalysts are synthesized by modifying TiO_2_ with selected additives: Y_2_O_3_, Ga_2_O_3_, and WO_3_. To achieve this, 1 g (10 Wt%) of each oxide is mixed with 10 mL of the initial TiO_2_ solution. Subsequently, 2.5 mL of polyethylene glycol (PEG), 5 mL of isopropanol, and 2 mL of acetylacetone are added to the mixture. This solution is then placed in a sonication machine for 1 h to obtain a coagulated texture. The coagulated mixture is then coated onto fluorine-doped tin oxide (FTO) glass (VWR International, Radnor, PA, USA) to facilitate potentiostat measurements. The coated glass is kept at room temperature and then heated in an oven at 250 °C for 2 h to evaporate all liquid components, yielding a thin film of photocatalyst firmly adhered to the FTO surface. WO_3_ was used as a commercialized product; however, Ga_2_O_3_ and Y_2_O_3_ were synthesized by precipitation and hydrothermal methods to obtain nanoscale oxides. All the conditions of the synthesis are described in the table below Table 1.

For the degradation test, diclofenac (Sigma Aldrich) and ketoprofen (Sigma Aldrich) were used as pharmaceutical pollutants.

### 2.2. Experimental Setup and Procedure

The experimental reactor was designed to facilitate the measurement of photocurrents and degradation within a single unit. It comprises a reactor vessel with a capacity of 145 mL, in which a catalyst coated onto FTO glass is fixed on one side, facing inward. A magnetic stirrer is installed at the reactor bottom to create turbulent conditions. Radiation is provided by an LED array consisting of 9 UV-A LEDs (Seoul, Republic of Korea, CUN66A1B) and 9 visible LEDs (Creed, Paris, France, XPCWHT-L1-0000-00C01). The UV-A LEDs have a peak wavelength of 365 nm and a maximum radiant flux of 1 W. The LED array is positioned parallel to the catalyst at a distance of 58 mm, ensuring that the radiation passes through the FTO glass to reach the catalyst. This setup delivers an incident UV-A radiation of 1222 W/m^2^ at maximum LED power.

To investigate the photocatalytic performance of TiO_2_ as a thin film and the effect of the addition of some oxides Y_2_O_3_, Ga_2_O_3_, and WO_3_ on fixed films, ketoprofen and diclofenac were selected as pharmaceutical pollutants for degradation studies at fixed concentration for each pollutant: 10 mg/L for KETO and 5 mg/L for DICLO. with immobilized photocatalysts as thin films based on TiO_2_ coated on a glass slide.

Supposing that the photocatalytic degradation of ketoprofen and diclofenac reaction follows a first-order kinetic described in the equation bellow [33]:

$$r=-\frac{dC}{dt}=\ln\left(\frac{C}{C_{0}}\right)=-Kt$$

where *r* is the reaction rate, *C* is the contaminant concentration during irradiation (mg/L), *C*_0_ is the initial concentration of contaminant, *K* is the rate constant of the first-order reaction (min^−1^), and *t* is the irradiation time (min). For the photocurrent measurement, the Rodeostat HC by IO Rodeo was used. As a counter electrode, a platinum wire was used. The reference electrode was a Ag/AgCl electrode. The photocurrent measurement was conducted using Raspberry Pi Model 4 and Python script controlling the potentiostat and the LED array. Both LED types were controlled separately using constant current sources and the PWM function of the Raspberry Pi. Within the code, the following steps were taken:

- Successive increase UV-LED power by 10% from 0 to 90% with a duration of 60 s:
  ○ Blinking of LED every 5 s (ON/OFF);
  ○ Continuous measurement of photocurrent.
- Successive increase VIS-LED power by 10% from 0 to 90% with a duration of 60 s:
  ○ Blinking of LED every 5 s (ON/OFF);
  ○ Continuous measurement of photocurrent.
- Successive increase UV- and VIS-LED power by 10% from 0 to 90% with a duration of 60 s:
  ○ Blinking of LED every 5 s (ON/OFF);
  ○ Continuous measurement of photocurrent.

The photocurrent measurement was conducted with the following potentiostat parameters:

- Constant voltammetry with 1.5 V;
- Sampling rate: 5 per second;
- Current range: 5000 µA.

The measurement of the photocurrents was evaluated by calculating the linear gradient of the photocurrents with a gradual (10%) increase in the illuminance of the 365 nm UV-A light

### 2.3. Analytical Methods

**Figure 1:**
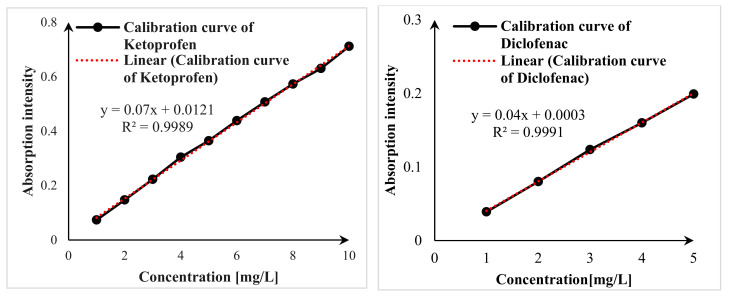
Calibration curves of ketoprofen and diclofenac.

The UV-Vis measurements were carried out using the Mettler Toledo UV5 Bio, Gießen, Germany at 365 nm. Figure 1 presents the calibration curve for ketoprofen and diclofenac standard solutions. The spectra display the dependence of absorption intensity on the mass concentration (mg/L) of both pharmaceutical molecules, demonstrating good linearity.

The reflectance measurements were carried out using an Ocean Optics USB 650 spectrometer between 350 and 800 nm with a reflection probe and a Halogen light source from Thunder Optics, Montpellier, France.

## 3. Results and Discussion

### 3.1. The Effect of the Oxide Addition on the Photocatalytic Properties of TiO_2_ as a Thin Fixed Film

In the first slide, only TiO_2_ was used, while the other slides included a 10 wt.% addition of Y_2_O_3_, Ga_2_O_3_, or WO_3_ relative to the mass of TiO_2_. At room temperature, the reactor was placed before nine UV LEDs (1 W/LED, radiometric output).

### 3.2. Measurement of the Photocurrent

**Figure 2:**
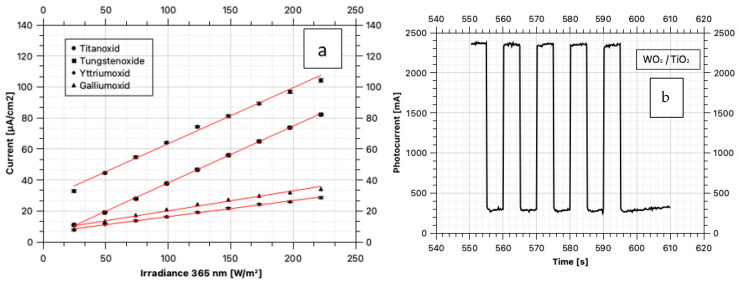
Photocurrents under different illuminance levels with different oxide mixtures (**a**); time sequence of the exposure during the measurements of the photocurrents (**b**).

The photocurrents were determined at increasing, equidistant illuminance levels with LEDs switched on and off at 365 nm monochromatic illumination. A linear correlation between the photon current and the resulting photocurrent was found for all oxides investigated (Figure 2). However, the individual samples differ significantly in the slope of the linear function (Table 2). Titanium dioxide and tungsten oxide show a very high similarity in the increase of the photocurrents, whereas the samples with gallium and yttrium oxide show a significantly lower gradient. No direct correlation between the increases in the photocurrents and the degradation rate of the tested substances could be determined in further observations, but a good possibility could be demonstrated to show the dependence of photocurrents on the photon density used and in future to determine the recombination effects with higher photon currents or to calculate the photon efficiency in relation to the free electrons released in the semiconductor.

### 3.3. SEM Results

**Figure 3:**
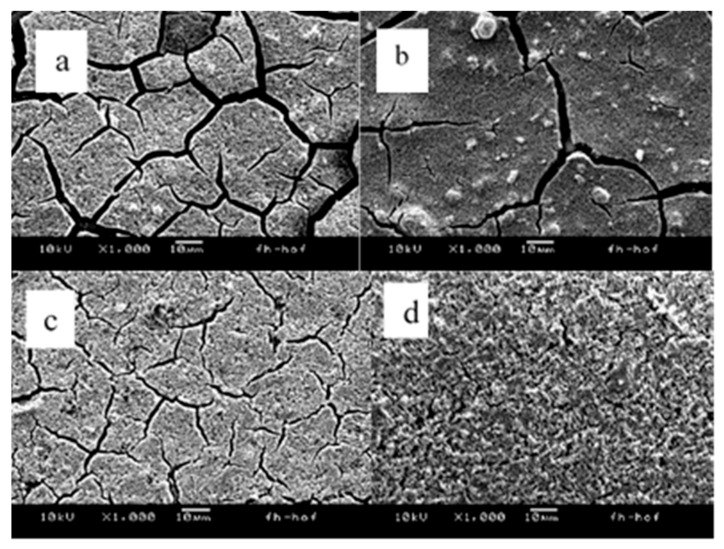
SEM images of photocatalyst slides: (**a**) TiO_2_, (**b**) TiO_2_/WO_3_, (**c**) TiO_2_/Ga_2_O_3_, and (**d**) TiO_2_/Y_2_O_3._

The homogeneity of the prepared photocatalysts TiO_2_/Y_2_O_3_, TiO_2_/Ga_2_O_3_, and TiO_2_/WO_3_ was analyzed using scanning electron microscopy (SEM). This analysis aimed to investigate the effect of the additives on the dispersion of TiO_2_ on the surface of the FTO glass. The SEM image in Figure 3a reveals that the TiO_2_ slide exhibits numerous wide cracks over an area of just 130 µm. However, the number of cracks significantly decreases in the same area dimension of the slide containing TiO_2_/WO_3_, as shown in Figure 3b. The SEM image of the photocatalyst TiO_2_/Ga_2_O_3_ in Figure 3c displays a homogeneous surface with small agglomerates and slight scratches, indicating that the addition of Ga_2_O_3_ to TiO_2_ enhances the uniformity and adhesion of the thin film to the glass support compared to the TiO_2_-only film. The SEM image of TiO_2_/Y_2_O_3_ in Figure 3d shows the highest degree of homogeneity among the combinations, with nanocomposites adhering to each other to form a uniform surface on the film. This suggests that the addition of Y_2_O_3_ nanoparticles effectively improves the dispersion of TiO_2_ on the FTO glass surface, resulting in a homogeneous photocatalyst with well-dispersed, fine grains.

### 3.4. The Effect of Doping with Other Oxides on the Band Gap of the Titanium Dioxide Base Material

**Figure 4:**
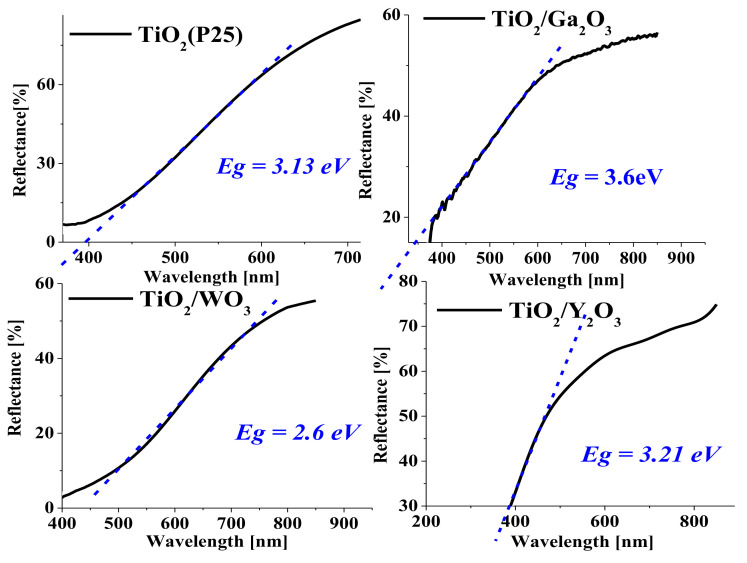
The effect of the additive oxides on the energy gap of the TiO_2_ base material.

This part aims to investigate the effect of adding the oxides WO_3_, Y_2_O_3_, and Ga_2_O_3_ on the energy gap (Eg) of TiO_2_ as a thin film by measuring the Eg of each prepared slide. The spectroscopy results, presented in Figure 4, demonstrate a shift in the Eg of each slide compared to that of pure TiO_2_. The spectra for the TiO_2_/Y_2_O_3_ and TiO_2_/Ga_2_O_3_ slides show slight differences in their Eg values (3.21 eV and 3.6 eV, respectively) compared to the Eg of TiO_2_ (3.13 eV) [34], indicating that these slides still primarily absorb UV light. However, the Eg values for the TiO_2_/WO_3_ slides (2.6 eV respectively) show significant deviations from the Eg of TiO_2_, corroborating previous studies with similar findings [35,36]. This shift confirms that the addition of these additives to TiO_2_ extends its absorption into the visible range (400–800 nm). Consequently, these modified slides can be utilized as photocatalysts under artificial white light or solar light, potentially enhancing energy efficiency.

### 3.5. The Effect of Additives on the Photocatalytic Efficiency of TiO_2_

#### 3.5.1. Photocatalytic Degradation of Ketoprofen

**Figure 5:**
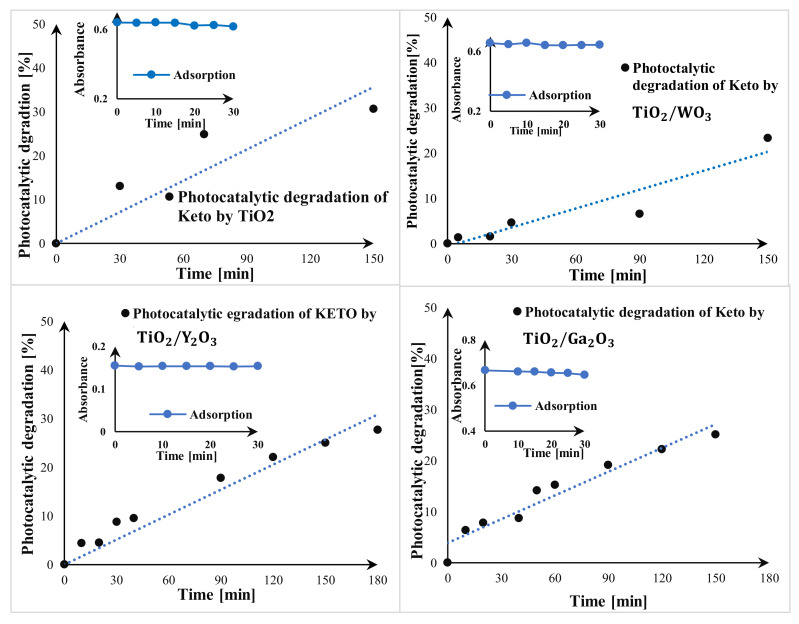
Study of ketoprofen photocatalytic degradation by TiO_2_; TiO_2_/WO_3_; TiO_2_/Y_2_O_3_; TiO_2_/Ga_2_O_3_.

The variations in the percentage of pollutant elimination over time are illustrated in Figure 5. The adsorption test conducted with only the photocatalysts, without UV light, resulted in zero elimination of KETO after 30 min. This indicates that adsorption does not affect the pollutant elimination process. However, in the presence of photocatalysts under UV LEDs, there is a noticeable increase in the percentage of KETO degradation during irradiation treatment. Photolytic degradation of diclofenac and ketoprofen at a wavelength of 365 nm can be ruled out, as this has already been investigated in a previous study with a higher radiometric power on the catalyst and no photolysis occurred [37]. This confirms the photocatalytic nature of the elimination reaction, wherein the particles of the thin film absorb UV radiation, activating the reactive nanoparticles on the film, which leads to the degradation of ketoprofen molecules in the solution.

The spectra of the TiO_2_ thin film demonstrated good efficiency in the photocatalytic degradation of KETO under UV LED light, achieving 30% degradation after 150 min. TiO_2_/Y_2_O_3_ and TiO_2_/Ga_2_O_3_ slides exhibited similar results to the TiO_2_-only slide, with a slight decrease, which suggests that these oxides do not significantly affect the photocatalytic degradation reactivity of TiO_2_ in the presence of the KETO molecule. This phenomenon can be attributed to a decrease in the number of active sites on the photocatalyst surface due to surface coating by the KETO molecules (10 mg/L) [38,39].

The TiO_2_/WO_3_ slide showed a 10% decrease in degradation efficiency compared to TiO_2_ results over the same treatment period, this observation can be explained by the previous reason) [38,39] and the shift of the energy gap of photocatalyst nearly to the visible light range as it demonstrated by the energy gap calculation.

#### 3.5.2. Photocatalytic Degradation of Diclofenac

**Figure 6:**
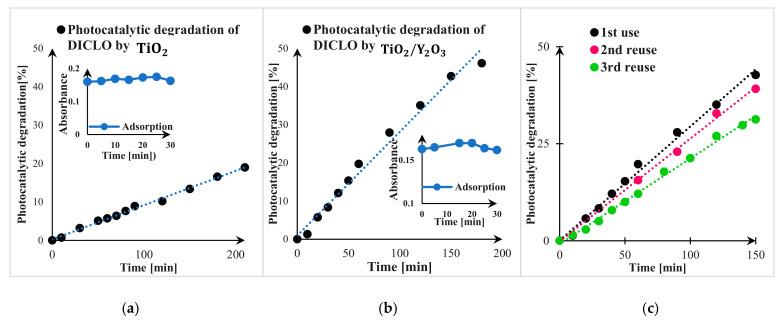
The effect of Y_2_O_3_ on TiO_2_ in photocatalytic degradation of diclofenac: (**a**) TiO_2_; (**b**) TiO_2_/Y_2_O_3_; (**c**) the reuse of TiO_2_/Y_2_O_3_.

The degradation of diclofenac using the prepared slides yielded different results compared to ketoprofen. As shown in the spectra in Figure 6a, the TiO_2_ slide exhibited no adsorption efficiency but demonstrated photocatalytic properties, degrading around 16% of diclofenac over 180 min. This degradation rate is insufficient to consider the TiO_2_ thin film an effective photocatalyst for diclofenac under UV LED light. Consequently, only TiO_2_/Y_2_O_3_ and TiO_2_/Ga_2_O_3_ slides were selected to investigate if they can enhance the efficiency of TiO_2_ with DICLO under the UV LED light.

TiO_2_/Ga_2_O_3_ showed 0% elimination of diclofenac, even when using a new slide for each experiment. The results were unexpected for the TiO_2_/Ga_2_O_3_ photocatalyst, which has an energy gap of 3.6 eV. This indicates that adding Ga_2_O_3_ to TiO_2_ makes the prepared thin film selective, effectively degrading ketoprofen but not reacting with diclofenac.

However, the TiO_2_/Y_2_O_3_ slide achieved a diclofenac degradation of approximately 46% over 180 min, which is three times better than TiO_2_ alone. This aligns with other studies that indicate single-component photocatalysts generally have a slower degradation rate than modified ones combined with other semiconductors, noble metals, or carbon composites [40]. With a small concentration of DICLO 5 mg/L, the active sites were more available on the surface of the photocatalyst, which leads to high absorption of UV light causing an increase in $O_{2}^{.-}$ and ${OH}^{.}$ radical generation, which can enhance the photocatalytic degradation of DICLO. Additionally, the nanoparticle size of Y_2_O_3_ increases the specific surface area of the photocatalyst, providing more active sites for redox reactions and generating more electron-hole pairs [41].

Moreover, the TiO_2_/Y_2_O_3_ slide proved reusable, maintaining effectiveness with a slight decrease in photocatalytic degradation percentage over three cycles: 42%, 40%, and 32% over 150 min, as shown in Figure 6c. This demonstrates that the synergistic properties of Y_2_O_3_/TiO_2_ create a more effective and reusable photocatalyst for degrading the organic molecule diclofenac. 

### 3.6. Kinetic Study of Ketoprofen and Diclofenac Degradation

The results confirmed that the kinetic photocatalytic degradation of both ketoprofen and diclofenac with all slides follows a first-order kinetic, as shown in Table 3. The reaction constant of ketoprofen degradation shows similar values of ca. 0.002 1/min for all catalyst variants investigated. However, for diclofenac degradation, significant differences in degradation rates are evident. For the sole TiO_2_ catalyst, a half-time value of 755 min was calculated. In contrast, the TiO_2_/Y_2_O_3_ catalyst achieved a half-time value of 215 min, representing an increase in reaction efficiency of more than 300%. 

## 4. Conclusions

This work investigated the influence of the additives Y_2_O_3_, Ga_2_O_3_, and WO_3_ on the photocatalytic degradation of pharmaceuticals by TiO_2_-based immobilized catalysts under UV-A irradiation. All additives showed influences on the surface structure of the prepared catalyst. For WO_3_ reflectance measurements indicated the influence of the catalysts band gap. The degradation test revealed the substance-specific behavior of the photocatalytic degradation, which was significantly influenced by all additives. For ketoprofen, reaction rates of a similar order of magnitude were determined, whereby the case values were determined for TiO_2_ without additives. The degradation of diclofenac was almost completely hindered by Ga_2_O_3_, and WO_3_ additives, whereby the addition of Y_2_O_3_ more than tripled the degradation rate compared to the sole TiO_2_ catalyst. In addition to the shifted band gap, which enables better conversion of the photons at the photocatalyst and the resulting higher production of hydroxyl radicals, this circumstance can be explained by the much more homogeneous structure with smaller particle size visible in the SEM images and thus a higher surface area for the reaction. Furthermore, the catalysts with yttrium oxide are less brittle and showed no stress cracks, which should improve the mechanical stability of the catalyst. The substance-specific nature observed indicates an influence of investigated additives on the overall adsorption characteristics of the composite catalysts. This effect could be utilized to improve the photocatalytic degradation of specific pollutants in complex matrices or in the context of photocatalytic sensors.

## Figures and Tables

**Table 1:** Synthesis of Ga_2_O_3_ and Y_2_O_3_ nanorods.

	Ga_2_O_3_	Y_2_O_3_
Precursors	2.6 g of 99.9% Ga(NO_3_)_3_·nH_2_O (Sigma-Aldrich) 100 mL Deionized Water NH_4_OH	99.9% Y(C_2_H_3_O_2_)_3_·4H_2_O (Alfa Aesar, Haverhill, MA, USA) 12 mL Deionized Water (0.3 M) 96% NaOH
Reached pH	9	13
Method synthesis	Precipitation (95 °C/5 h) Calcination (1000 °C/3 h)	Hydrothermal (200 °C/2 h and then 180 °C/5 h) Calcination (1000 °C/3 h)
Treatment of recovered product	Filtration Washing with DW Drying 80 °C/5 h	Centrifugation 6000 rpm/15 min Washing with DW Drying 80 °C/10 h
Product before calcination	GaOOH nanorods	Y(OH)_3_
Final product	β-Ga_2_O_3_ nanorods	Y_2_O_3_ nanorods

**Table 2:** Linear function of the photocurrent to the irradiation.

Oxide Phase	Slope (µA/W/cm^2^)/(W/m^2^)	R^2^
TiO_2_	0.361	0.999
TiO_2_/WO_3_	0.366	0.999
TiO_2_/Y_2_O_3_	0.128	0.998
TiO_2_/Ga_2_O_3_	0.103	0.996

**Table 3:** Degradation constant, half-time value, and R^2^ of investigated catalysts for diclofenac and ketoprofen degradation.

Catalyst	Pollutant	k [1/min]	Half-Time [min]	R^2^ [-]
TiO_2_	DICLO	0.000918	755.0	0.993
TiO_2_/Y_2_O_3_	DICLO	0.003224	214.9	0.980
TiO_2_	KETO	0.002941	235.6	0.845
TiO_2_/Y_2_O_3_	KETO	0.001987	348.7	0.965
TiO_2_/Ga_2_O_3_	KETO	0.002235	310.0	0.883
TiO_2_/WO_3_	KETO	0.001740	398.2	0.989

## Data Availability

The original contributions presented in the study are included in the article, further inquiries can be directed to the corresponding author.
